# The complete chloroplast genome sequence of *Lespedeza buergeri* Miq. (Fabaceae)

**DOI:** 10.1080/23802359.2022.2093674

**Published:** 2022-07-06

**Authors:** Shuting Wang, Fei Meng, Jing Wu, Xiaoyan Yao, Xiaohu Guo, Liping Wu, Jing Zhang, Shihai Xing

**Affiliations:** a College of Pharmacy, Anhui University of Chinese Medicine, Hefei, China; b Institute of Traditional Chinese Medicine Resources Protection and Development, Anhui Academy of Chinese Medicine, Hefei, China; c Anhui Province Key Laboratory of Research & Development of Chinese Medicine, Hefei, China

**Keywords:** *Lespedeza buergeri;* Fabaceae, chloroplast genome, phylogenetic analysis

## Abstract

The complete chloroplast genome sequence of *Lespedeza buergeri* is presented in this report. It is 149,065 bp in length and divided into four distinct regions: a small single copy (SSC) region of 18,934 bp, a large single copy (LSC) region of 82,476 bp, and a pair of inverted repeat regions of 23,826 bp. The annotation of the *L. buergeri* complete chloroplast genome predicted a total of 123 genes (77 protein-coding genes, 38 transfer RNA genes, and 8 ribosomal RNA genes). Phylogenetic analysis with the reported chloroplast genomes revealed that *L. buergeri* is nested in the genus *Lespedeza* of Fabaceae family. Furthermore, *L*. *buergeri* exhibited a close relationship with *Lespedeza bicolor* and *Lespedeza cuneata*. This results in this study might contribute to further investigating the evolutionary relationship of family Fabaceae.

*Lespedeza buergeri* Miq.1876 belongs to the genus Lespedeza, and this genus contains about 60 species within the family Fabaceae (Sun et al. 2021). *L. buergeri* is a shrub disjunctively distributed in China, Korea, and Japan (Jin et al. 2016). *L. buergeri* commonly grows in forest or forest slopes above 500 m above sea level and is primarily planted in the greening of highways and railway slopes due to its strong drought resistance (Zhang et al. 2021). However, it does not only give full play to the ornamental value, such as long flowering period, rich flower color, and beautiful posture, but also the leaves and roots of *L. buergeri* have medicinal values (Jin et al. 2016; Sun et al. 2021). In this study, the complete chloroplast (Cp) genome sequence of *L. buergeri* was reported to provide a genomic resource and elucidate the phylogenetic relationship between this plant and other species in the Fabaceae family and other related plants. The results can contribute to a better understanding of the phylogenetic position of the species and provide important genetic information for further research. The sequence was submitted to GenBank with the accession number OM214533.

The fresh young leaves of *L. buergeri* were collected from Anhui University of Chinese Medicine (117°38'E, 31°93'N), and the specimen was deposited in the Center of Herbarium, Anhui University of Traditional Chinese Medicine, Hefei, China, under accession number 20211019 (AhtcmH, yxy.ahtcm.edu.cn/info/1006/6713.htm, Shi-hai Xing, xshshihai@163.com). Genomic DNA was extracted by DNAsecure Plant Kit (Tiangen Biotech Co., Ltd., Beijing, China). A total of 200 μg of genomic DNA was randomly fragmented to an average size of 300–350 bp, and the obtained DNA was constructed into paired-end (PE) libraries of an average of 500 bp. DNA libraries with different indices were multiplexed and loaded on an Illumina HiSeq instrument according to manufacturer’s instructions (Illumina, San Diego, CA, USA). Sequencing was carried out using a 2 × 150 paired-end configuration, image analysis and base calling were conducted by the HiSeq control software (HCS) + OLB + GAPipeline-1.6 (Illumina) on the HiSeq instrument. The reads were quality controlled and then assembled using velvet (version 1.2.10), gaps filled with SSPACE (version 3.0) and GapFiller (version 1–10) (Zerbino and Birney 2008; Boetzer et al. 2011; Boetzer and Pirovano 2012). Based on the clean data, the chloroplast genome of *L. buergeri* was assembled using the software NOVOPlasty 2.7.2 (Dierckxsens et al. 2017) and auxiliary software Spades (Bankevich et al. 2012) on all the contigs, which used the complete Cp genomic sequences of *L. bicolor* (GenBank: NC_046836) was utilized as a reference genome for predicting genes by Prodigal (version 3.02) (Hyatt et al. 2010).

The Cp genome of *L. buergeri* was a typical circular form of 149,065 bp in length and was separated into four distinct regions: a large single copy (LSC) region of 82,476 bp, a small single copy (SSC) region of 18,934 bp, and a pair of inverted repeat regions of 23,826 bp. Overall, the GC content of this Cp genome was 35.82%. The Cp genome of *L. buergeri* encoded a total of 123 genes, which belong to three categories, including 77 protein coding genes, 8 ribosomal RNA (rRNA) genes and 38 transfer RNA (tRNA) genes. The assembled complete Cp genome sequences of *L. buergeri* had been submitted to NCBI.

Furthermore, the Cp genome sequences of 21 species were aligned by MAFFT v7 to understand the phylogenetic relationship between *L. buergeri* and other related species (Katoh and Standley 2013). Afterward, the evolutionary history was inferred by using the maximum-likelihood (ML) approach in MEGA7.0 (Kumar et al. 2016) in the Tamura-Nei substitution model (Kumar et al. 2018). Bootstrap (BS) value was calculated through 1000 times of repeated analyses (Stamatakis et al. 2008) (Figure 1). As expected, *L. buergeri* closely grouped with *L. bicolor* and *L. cuneata* in genus *Lespedeza*. This complete Cp genome can contribute to future population genomic studies, DNA barcoding, and conservation genetics.

**Figure 1.**
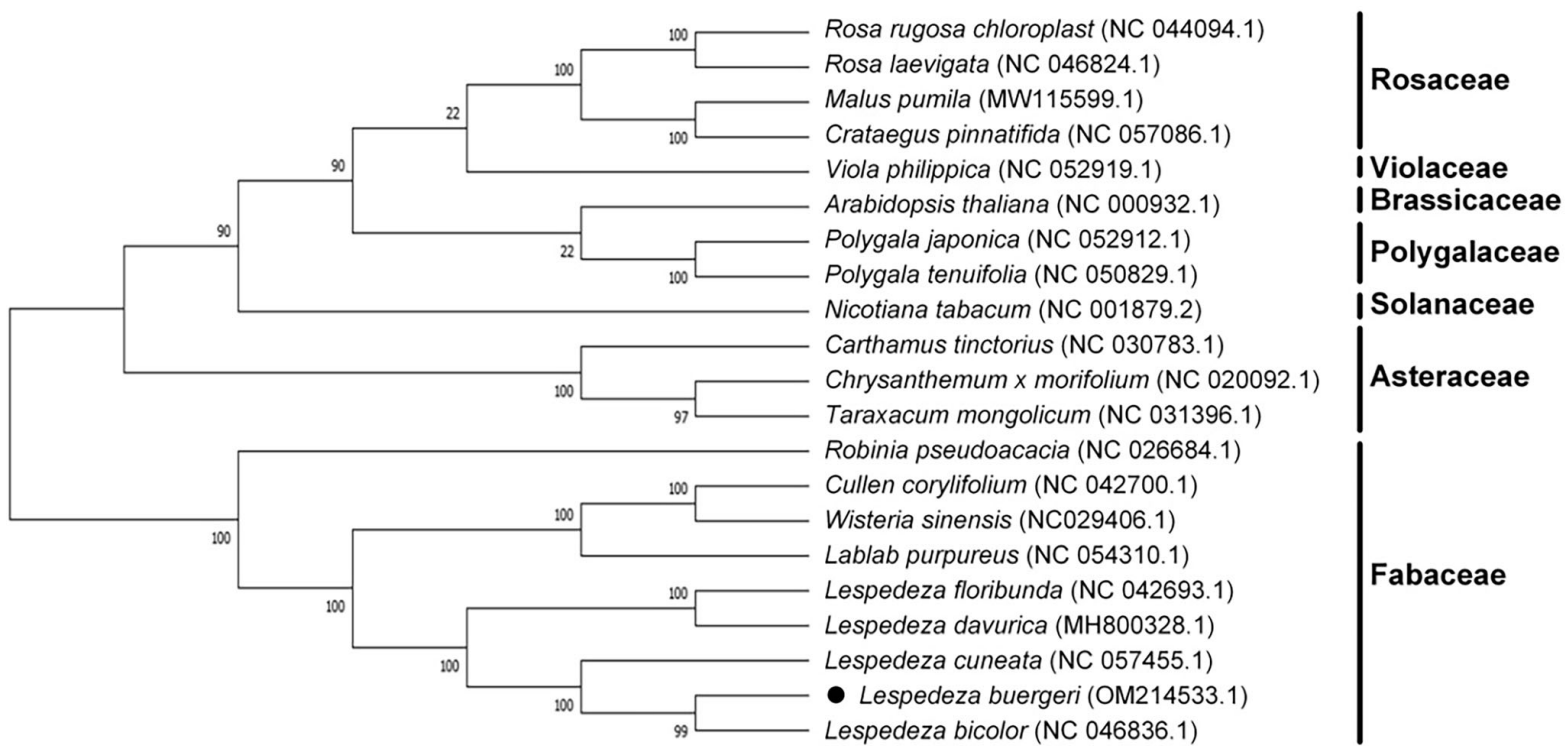
Phylogenetic tree plotting by Maximum Likelihold Method based on an alignment of the completed chloroplast genome sequences of *Lespedeza buergeri* Miq. and 20 other representative species. The bootstrap parameter was set as 1,000 replicates.

## Data Availability

The data that support the findings of this study are openly available in GenBank of NCBI (https://www.ncbi.nlm.nih.gov) under the access number OM214533. The other data such as associated BioProject, SRA and Bio-Sample numbers are PRJNA797562, SRR17633451, and SAMN25013315, respectively.
